# Silent uterine rupture in the term pregnancy: Three case reports

**DOI:** 10.1097/MD.0000000000037071

**Published:** 2024-03-08

**Authors:** Lei Chen, Hao Li, Jing Peng, Min Li, Ying Wang, Kai Zhao, Lijun Yang, Yun Zhao

**Affiliations:** a Department of Obstetrics, Maternal and Child Health Hospital of Hubei Province, Tongji Medical College, Huazhong University of Science and Technology, Hongshan District, Wuhan, China; b Institute of Reproductive Health, Tongji Medical College, Huazhong University of Science and Technology, Qiaokou District, Wuhan, China.

**Keywords:** asymptomatic, induced abortion, laparoscopic myomectomy, laparoscopic transabdominal cerclage, uterine rupture

## Abstract

**Rationale:**

Uterine rupture is extremely hazardous to both mothers and infants. Diagnosing silent uterine rupture in pregnant women without uterine contractions is challenging due to the presence of nonspecific symptoms, signs, and laboratory indicators. Therefore, it is crucial to identify the elevated risks associated with silent uterine rupture.

**Patient concerns:**

on admission, case 1 was at 37 gestational weeks, having undergo laparoscopic transabdominal cerclage 8 months prior to the in vitro fertilization embryo transfer procedure, case 2 was at 38 4/7 gestational weeks with a history of 5 previous artificial abortion and 2 previous vaginal deliveries, case 3 was at 37 6/7 gestational weeks with a history of laparoscopic myomectomy.

**Diagnoses:**

The diagnosis of silent uterine rupture was based on clinical findings from cesarean delivery or laparoscopic exploration.

**Interventions:**

Case 1 underwent emergent cesarean delivery, revealing a 0.25 cm × 0.25 cm narrow concave area above the Ring Ties with active and bright amniotic fluid flowing from the tear. Case 2 underwent vaginal delivery, and on the 12th postpartum day, ultrasound imaging and magnetic resonance imaging revealed a 5.8 cm × 3.3 cm × 2.3 cm lesion on the lower left posterior wall of the uterus, and 15th postpartum day, laparoscopic exploration confirmed the presence of an old rupture of uterus. Case 3 underwent elective cesarean delivery, revealing a 3.0 cm × 2.0 cm uterine rupture without active bleeding at the bottom of the uterus.

**Outcomes:**

The volumes of antenatal bleeding for the 3 patients were approximately 500 mL, 320 mL, and 400 mL, respectively. After silent uterine ruptures were detected, the uterine tear was routinely repaired. No maternal or neonatal complications were reported.

**Lessons:**

Obstetricians should give particular consideration to the risk factors for silent uterine rupture, including a history of uterine surgery, such as laparoscopic transabdominal cerclage, laparoscopic myomectomy, and induced abortion.

## 1. Introduction

Uterine rupture can lead to significant morbidity and mortality in both women and newborns. Most uterine ruptures occur in pregnant women.^[1]^ This condition manifests in 2 primary ways: uterine dehiscence and uterine rupture. Uterine dehiscence is characterized by incomplete division of the uterus that does not penetrate all 3 layers: the endometrium, myometrium, and perimetrium. Often, uterine dehiscence remains unnoticed and discovered incidentally in asymptomatic patients who exhibit stable fetal heart rate tracing. In contrast, uterine rupture involves complete division of all 3 layers of the uterus. Recently, there has been a growing incidence of uterine rupture driven by aspiration to extend the option of a trial of labor after cesarean section to a broader range of patients. The rupture can come to light during cesarean delivery or arouse suspicion through obstetric ultrasound. Regularly evaluating the scar in the lower uterine segment of women with a history of prior cesarean section using accessible ultrasound resources could prove to be beneficial.^[2]^ Uterine rupture can sometimes occur silently without any apparent signs, which is an uncommon event. The accidental discovery of such silent ruptures has rarely been documented.

## 2. Case reports

In this context, we present 3 cases of silent uterine rupture in which the rupture was asymptomatic and the patients remained hemodynamically stable at our birth center.

Ethical approval was not considered necessary as this was a retrospective study, and all data were obtained solely from patients’ medical records and follow-ups via telephone and WeChat. All women included in this study signed informed consent for both therapeutic procedures and the publication of the case report.

### 2.1. Case 1

A 39-year-old primiparous woman, gravida 4, para 0, was admitted to our Labor and Delivery Department because of reduced fetal movement, lack of reaction in the nonstress test (NST), and pregnancy hypertension (153/85 mm Hg) at 37 weeks of gestation. The current pregnancy resulted from first-generation in vitro fertilization embryo transfer (IVF-ET). Her body mass index before pregnancy and on admission was 24.2 kg/m^2^ and 28.0 kg/m^2^, respectively. During the course of the current pregnancy, she received regular prenatal care 10 times. Screening for gestational diabetes mellitus (GDM) yielded negative results, and the estimated birth weight on admission under ultrasound was approximately 3200 g. She had a history of medical termination of pregnancy due to a fetal abnormality at 22 weeks of gestation using mifepristone combined with Ethacridine Lactate, followed by intracardiac KCl administration. Subsequently, she had a single twin pregnancy, both of which required termination through IVF-ET at 19 weeks of gestation. For each termination in the second trimester, the patient underwent dilation and curettage 3 times because of a retained placenta. As a result, she opted for laparoscopic endocervical cerclage using Mousseline Ring Ties 8 months prior to the current IVF-ET procedure at our hospital. As a result, she opted for laparoscopic endocervical cerclage using Mousseline Ring Ties at our hospital before undergoing IVF-ET. On admission, there was no tenderness in the abdominal region. During vaginal examination, the fetal head was positioned high, the cervical os showed no dilation, and the cervical length was approximately 2 cm.

Upon admission, an emergency cesarean delivery was immediately performed because of fetal distress and pregnancy-induced hypertension. The procedure was performed under combined spinal-epidural anesthesia. An abdominal incision was made transversely above the suprapubic region. Upon opening the peritoneum, it was observed that the lower part of the anterior uterine wall, near the bladder peritoneal reflection, had a narrowing concave-shaped tear measuring 0.25 cm × 0.25 cm. The tear was positioned just above the ring’s ties. Notably, the clear amniotic fluid gradually emanated from the concave tear (See video, Supplemental video, Supplemental Digital Content 1, http://links.lww.com/MD/L396, which demonstrates the amniotic fluid emanated from the concave tear). A transverse incision in the lower uterine segment was selected for delivery. The newborn, a girl weighing 3270 g, had Apgar scores of 10 at 1 minute and 10 at 5 minutes. The placenta was routinely extracted and exhibited no pathological signs. Neither the bladder nor the internal genital organs showed any pathological findings. Subsequently, the ring ties were removed and the tear was meticulously repaired using Vicryl 2/0. Following fetal removal, cefazolin antibiotic treatment was initiated. In response to uterine atony, standardized dosages of oxytocin and cartoprost tromethamine were administered. Overall, the estimated blood loss during the surgery was approximately 500 mL.

### 2.2. Case 2

**Figure 1.**
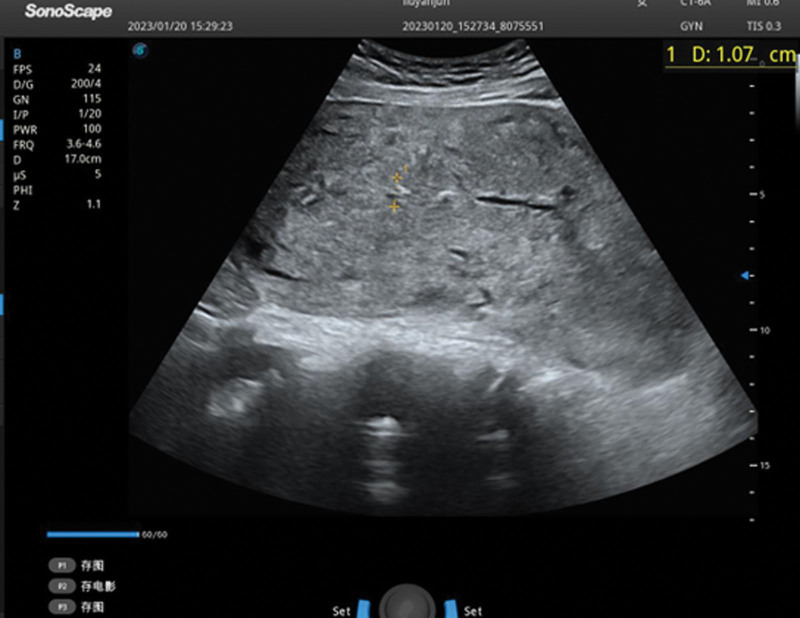
Three days postpartum, no obvious abnormality of the uterus or adnexa confirmed by ultrasound examination.

A 39-year-old multiparous woman, gravida 8, para 2, was admitted to our Labor and Delivery Department at 38 4/7 gestational weeks due to persistently elevated blood glucose levels over a duration of 3 months. She had a history of 2 spontaneous vaginal deliveries and 5 induced abortions. At 24 weeks of gestation, her oral glucose tolerant test results were 4.76/7.33/9.43 mol/L, leading to a diagnosis of GDM. She attempted to control her blood sugar through diet and exercise but with poor results. Shortly after admission, she opted for labor induction with 0.5% oxytocin, with a Bishop score of 6. Four hours later, she delivered a baby boy weighing 3550 g who subsequently experienced a first-degree perineal tear. The Apgar scores were 10/1 minute and 10/5 minutes, respectively. The placenta was expelled naturally, accompanied by a hemorrhage of 320 mL during labor and 2 hours after delivery. Her recovery was uneventful, and she was discharged 3 days postpartum with no observable abnormalities in the uterus or adnexa, as confirmed by ultrasound examination (Fig. 1).

**Figure 2.**
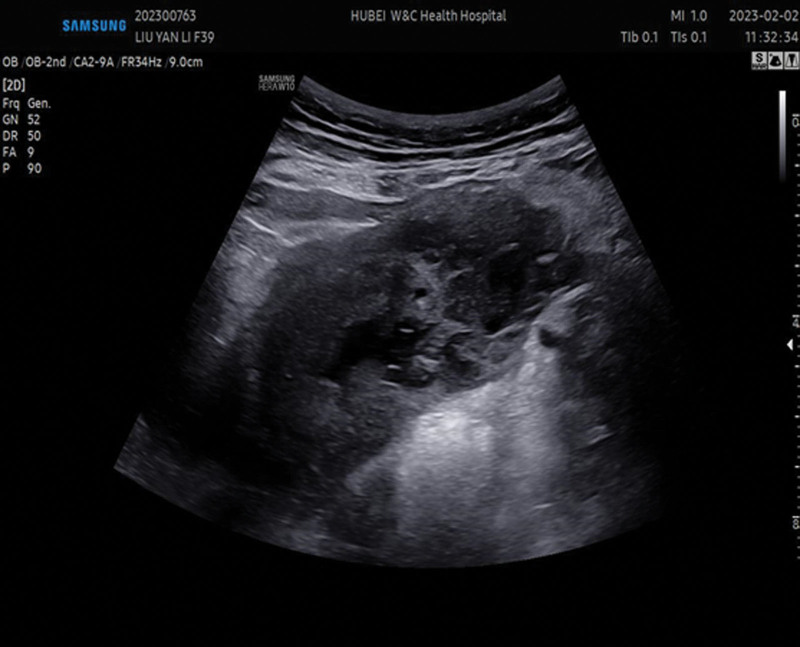
The ultrasound revealed a heterogeneous echogenic area on the lower left posterior uterine wall.

**Figure 3.**
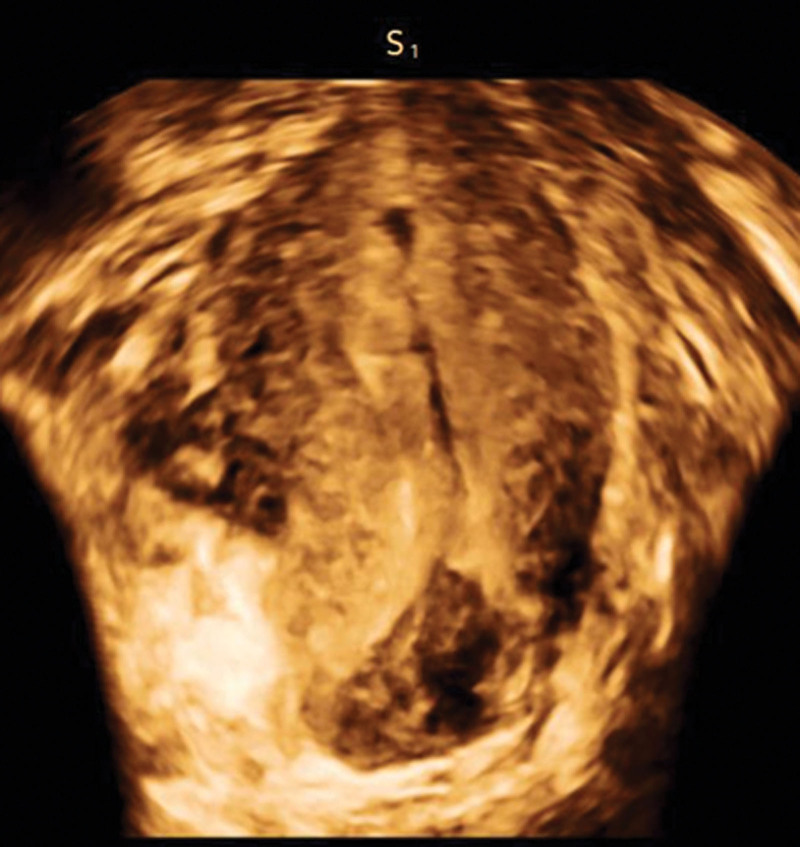
Enhanced ultrasound imaging.

**Figure 4.**
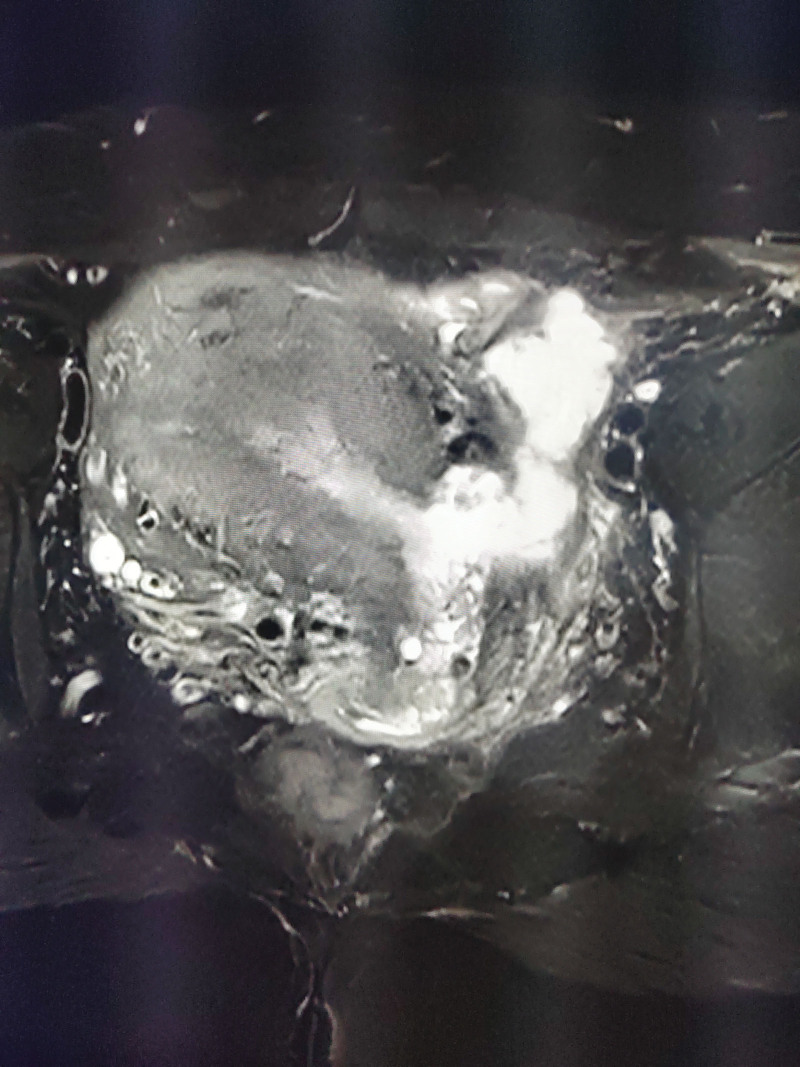
On MRI, the lesion appeared as a localized mass and was also found to be linked to the uterine cavity, with evidence of myometrial discontinuity. MRI = magnetic resonance imaging.

Twelve days postpartum, the patient experienced persistent lower abdominal and lower back pain, particularly noticeable with changes in posture. Physical examination revealed pressure sensitivity and rebound tenderness on the left side of the lower uterus. Subsequently, ultrasound and magnetic resonance imaging (MRI) were performed in the outpatient department. The ultrasound revealed a heterogeneous echogenic area measuring 5.8 cm × 3.3 cm × 2.3 cm on the lower left posterior uterine wall, extending towards the parietal aspect of the uterus (Fig. 2). Enhanced ultrasound imaging revealed contrast enhancement within the lesion, which was connected to the uterine cavity with well-defined peripheral borders (Fig. 3). On MRI, the lesion appeared as a localized mass with a discernible wall exhibiting irregular long T1 and T2 signal shadows, indicative of dispersion, and mild enhancement was noted on the enhanced scan. The lesion was also linked to the uterine cavity, with evidence of myometrial discontinuity (Fig. 4). Based on the combination of symptoms, clinical signs, and ultrasound and MRI findings, the lesion was diagnosed as an infectious lesion associated with uterine rupture.

After 3 days of conservative antibiotic treatment with levofloxacin and cefadroxil succinate sodium, the patient’s abdominal pain did not improve significantly. Subsequently, the patient underwent laparoscopic exploration at 15 days postpartum. Under general anesthesia, a firm mass measuring approximately 5 × 4 × 3 cm was discovered between the anterior and posterior lobes of the left broad ligament. The surface of the mass was intact, and rupture was evident. After opening the left posterior lobe of the broad ligament, the abscess cavity was visible. The wall of the cavity was firm and connected to the left cervical isthmus and the lateral vaginal fornix. The mass appeared indistinct from the surrounding tissue, and alignment of the left ureter was challenging to discern. The mass was dissected from the surrounding tissue, routine sutures were applied for hemostasis, and closure of the uterine wound and left vaginal vault was performed. Finally, the left ureteral stent was inserted under cystoscopic guidance. Intraoperative bleeding volume was estimated to be approximately 100 mL.

### 2.3. Case 3

**Figure 5.**
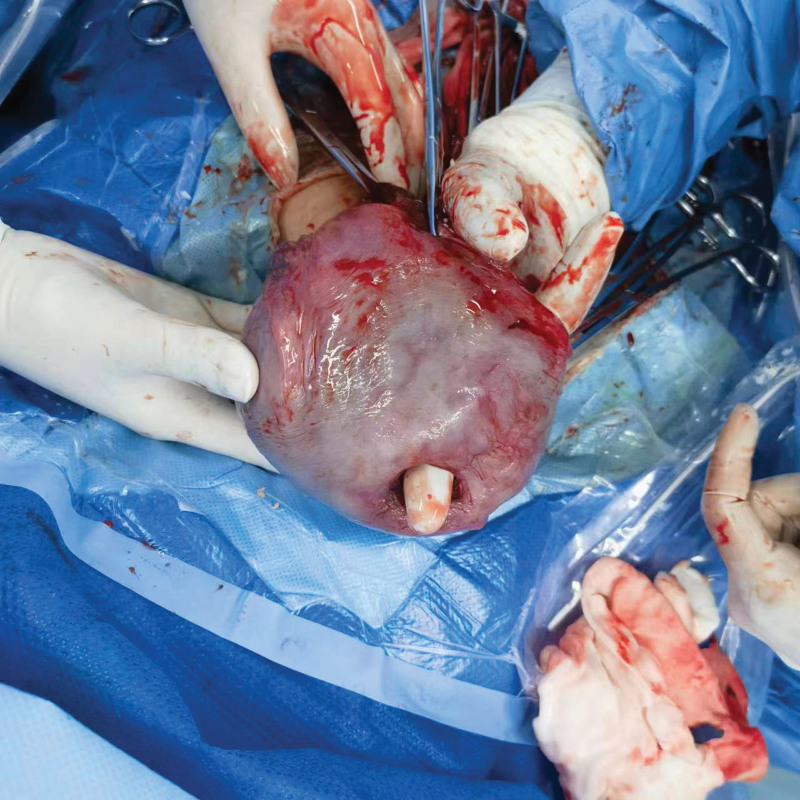
The rupture was connected to the uterine cavity.

A 43-year-old multiparous woman was admitted to our department at 37 6/7 gestational weeks due to polyhydramnios. She was a carrier of alpha-thalassemia and had undergone regular prenatal examinations a total of 13 times, experiencing a smooth pregnancy. Screening for GDM yielded negative results, and the estimated birth weight was approximately 4051 g on ultrasound. Sonographic evaluation revealed an amniotic fluid depth of 7.9 cm and an amniotic fluid index of 28.8 cm. She had a history of cesarean delivery 20 years prior to the current pregnancy at maternal request. Additionally, she had undergone laparoscopic myomectomy measuring 5cm × 5cm × 5 cm from the bottom of the uterus 2 years before the current pregnancy. Her BMI was 23.7 kg/m^2^ before pregnancy and 28.9 kg/m^2^ on admission. The patient’s initial vital signs were normal, and pelvic examination revealed a closed cervix. She underwent elective cesarean delivery 1 day after admission, with indications including fetal macrosomia, a history of previous cesarean section, a scarred uterus, and breech presentation. Under spinal combined with epidural anesthesia, an incision was made transversely above the suprapubic region to remove the previous scar. A transverse incision in the lower uterine segment was made for delivery. The newborn girl, weighing 3980 g, received Apgar scores of 10 at 1 minute and 10 at 5 minutes. The placenta was completely expelled. The amniotic fluid was clear and was estimated to be approximately 2000 mL. To induce uterine contractions, 20 units of oxytocin and 250 µg of carboprost tromethamine were administered to the uterine body. Routine suturing of the lower uterine incision was then performed. Following the procedure, the uterus and adnexa were examined, revealing a rupture approximately 3.0 cm × 2.0 cm in size at the bottom of the uterus. The rupture was connected to the uterine cavity and the tissue exhibited brittleness (Fig. 5). The rupture was repaired according to protocol. The intraoperative bleeding volume was approximately 400 mL. The patient recovered completely after the procedure and was discharged in a stable condition on the 6th postpartum day.

## 3. Discussion

Spontaneous uterine rupture is an infrequent and potentially severe obstetric complication linked to maternal mortality, fetal demise, postpartum hemorrhage, hemoperitoneum, substantial pelvic hematoma, and the potential necessity for cesarean hysterectomy if not promptly diagnosed and treated.^[3,4]^ The diagnosis of uterine rupture, which can sometimes be “clinically silent” or without obvious symptoms, can present particular challenges.^[5]^ The most prevalent causes of uterine rupture are widely recognized to include prior laparotomic myomectomy followed by subsequent cesarean section,^[5,6]^ previous cesarean section,^[7]^ a short interpregnancy interval or brief interval between myomectomy and conception (i.e. <12 months),^[8]^ abnormal placentation, placenta previa and percreta,^[9]^ or surgical termination of pregnancy.^[4]^ Nevertheless, cases of uterine rupture have also been documented in primigravida patients without evident risk factors.^[10]^ As such, it is imperative to maintain awareness of the potential for uterine rupture in all pregnant women.

Cervical insufficiency is a well-established cause of morbidity and mortality in newborns.^[11]^ Cerclage is a recognized therapeutic intervention for addressing recurrent spontaneous miscarriages or preterm births attributed to cervical insufficiency.^[12–14]^ Laparoscopic transabdominal cerclage has emerged as a highly effective and well-tolerated surgical option in selected patients.^[11]^ The approach is flexible, and the method chosen depends on various factors, including gestational age, technical feasibility, and surgeon’s expertise. For patients with an in situ transabdominal cerclage, cesarean delivery is recommended between 37 0/7 and 39 0/7 gestational weeks.^[15]^ Dandapani et al^[16]^ reported the experience of a woman with a transabdominal cerclage who was admitted at 39 2/7 gestational weeks, presenting with contractions, abdominal pain, and fetal bradycardia. Emergency cesarean delivery revealed a ruptured uterus with the fetus and placenta floating in the abdominal cavity. The Apgar scores were 2/1 minute, 2/5 minutes, and 5 minutes, respectively. The patient’s baby recovered successfully after neonatal rehabilitation.^[16]^ The most frequently observed initial sign of uterine rupture is the presence of nonreassuring fetal status patterns.^[7]^ In our study, case 1 involved a patient who underwent laparoscopic transabdominal cerclage due to 2 prior spontaneous abortions occurring at 19 weeks of gestation. After cerclage, the patient underwent IVF-ET and was successfully treated. At 37 weeks of gestation, she was admitted for reduced fetal movements, nonreaction to nonstress test, and pregnancy hypertension (153/85 mm Hg). An investigation revealed a small rupture measuring 0.25 cm × 0.25 cm, accompanied by active amniotic fluid above the site of the ring ties. This silent rupture can be attributed to 2 factors. First, the inelastic nature of the ring ties might hinder expansion, as the uterus undergoes spontaneous contractions in the later stages of pregnancy. Additionally, considering the patient’s history of dilation and curettage, it is possible that the tear was linked to these previous surgical interventions.

In an unscarred uterus resulting from a cesarean delivery or myomectomy, the risk of uterine rupture is lower. Notably, the risk factors for uterine rupture in an unscarred uterus include high parity.^[17]^ In case 2, concealed uterine rupture was identified 15 days postpartum due to persistent lower abdominal and low back pain, along with the discovery of a lump measuring 5.8 cm × 3.3 cm × 2.3 cm through ultrasound and MRI. The lump was identified as an abscess with an old and hardened wall. The abscess cavity was connected to the left cervical isthmus and lateral vaginal area. From this observation, we inferred that the silent rupture could be attributed to a former uterine laceration that likely occurred during 1 of the patient’s 5 previously induced abortions and was possibly followed by self-healing. In the current pregnancy, the old wound was gradually reopened. Shortly after delivery, continuous blood flow from the uterine bleeding passed through the reopened wound into the left broad ligament, resulting in abscess formation.

Uterine fibroids, the most common benign neoplasm of the female genital tract, may require myomectomy in women of childbearing age, who experience abnormal vaginal bleeding, pelvic pain, pressure symptoms, or infertility.^[18]^ Individuals with a history of myomectomy are at increased risk of uterine rupture in subsequent pregnancies.^[19]^ Most practitioners recommend elective cesarean delivery if the endometrial cavity is breached, if there is a short interval between surgery and pregnancy, or if there are large myomas (maximum diameter > 4 cm).^[8,20,21]^ Therefore, the description of surgical procedures should encompass the crucial elements relevant to subsequent pregnancies.^[20]^ Obstetricians are cautious in recommending cesarean deliveries to pregnant patients with a history of myomectomy. Myomectomy was identified as an independent factor contributing to uterine rupture, magnifying the risk by 14 times.^[22]^ The subsequent pregnancy rate was higher in individuals undergoing laparoscopic myomectomy than in abdominal myomectomy,^[23]^ although the incidence of uterine rupture during pregnancy was lower after abdominal myomectomy than after laparoscopic procedures.^[8]^ Hruban et al^[5]^ reported a 43-year-old tercigravida with a history of laparotomic myomectomy. In subsequent pregnancy, uterine rupture occurred at the site of the previous uterine scar. The fetus was entirely expelled into the abdominal cavity and the diagnosis was made at 24 6/7 gestational weeks. This case was concluded with elective cesarean section and hysterectomy at 28 gestational weeks due to the absence of clinical symptoms and satisfactory condition of the fetus. In our study, a patient (Case 3) with a history of laparoscopic myomectomy (5.0 cm × 5.0 cm × 5.0 cm) at the bottom of the uterus underwent elective cesarean delivery due to fetal macrosomia, previous cesarean section, scarred uterus, and breech presentation. The patient showed no overt signs of uterine rupture. However, during surgery, a 3.0 cm × 2.0 cm rupture without active bleeding was identified at the bottom of the uterus connecting to the uterine cavity. The concealed uterine rupture was directly associated with prior laparoscopic myomectomy performed to treat large myomas. The outcomes of uterine contractions may be consistent with those reported in previous studies.^[5,24]^

The rising incidence of uterine surgeries in clinical practice may lead to iatrogenic complications, potentially causing uterine damage and resulting in spontaneous uterine rupture during subsequent pregnancies. Diagnosing silent uterine rupture in pregnant women without uterine contractions is challenging because of the presence of nonspecific symptoms, signs, and laboratory indicators. However, uterine rupture is extremely hazardous to both mother and infant, especially when accompanied by active bleeding or infection. Obstetricians should give particular consideration to the risk factors for silent uterine rupture, including a history of uterine surgery, such as laparoscopic transabdominal cerclage, laparoscopic myomectomy, and induced abortion.

## 4. Limitation

While our conclusion is limited by the sample size, we believe that the findings of these 3 reports are valuable for understanding the possible clinical features of silent uterine rupture in pregnant women, as well as its treatment and prognosis. As our sample size was small, further multicenter research is needed to explore the risk factors for silent uterine rupture.

## Acknowledgments

The authors are grateful to the patients who provided informed consent for publication of this manuscript. This work was supported by the Foundation of Health and Family Planning Commission of Hubei Province (No. WJ2018H0133; No. WJ2019H297), and Public Safety Risk Prevention and Control and Emergency Technical Equipment (No. 2020YFC0860900).

## Author contributions

Conceptualization: Yun Zhao, Lei Chen, Hao Li.

Data curation: Jing Peng, Min Li.

Formal analysis: Ying Wang.

Funding acquisition: Yun Zhao.

Investigation: Hao Li.

Methodology: Lei Chen.

Project administration: Yun Zhao.

Supervision: Lijun Yang.

Writing – original draft: Hao Li.

Writing – review & editing: Hao Li, Kai Zhao.

## Supplementary Material


